# Deleted in Liver Cancer 1 (DLC1) Utilizes a Novel Binding Site for Tensin2 PTB Domain Interaction and Is Required for Tumor-Suppressive Function

**DOI:** 10.1371/journal.pone.0005572

**Published:** 2009-05-15

**Authors:** Lo-Kong Chan, Frankie Chi Fat Ko, Irene Oi-Lin Ng, Judy Wai Ping Yam

**Affiliations:** 1 Liver Cancer and Hepatitis Research Laboratory and SH Ho Foundation Research Laboratories, Department of Pathology, The University of Hong Kong, Pokfulam, Hong Kong, China; 2 Centre for Cancer Research, Li Ka Shing Faculty of Medicine, The University of Hong Kong, Pokfulam, Hong Kong, China; University of Birmingham, United Kingdom

## Abstract

**Background:**

Deleted in liver cancer 1 (DLC1) is a Rho GTPase-activating protein (RhoGAP) frequently deleted and underexpressed in hepatocellular carcinoma (HCC) as well as in other cancers. Recent independent studies have shown interaction of DLC1 with members of the tensin focal adhesion protein family in a Src Homology 2 (SH2) domain-dependent mechanism. DLC1 and tensins interact and co-localize to punctate structures at focal adhesions. However, the mechanisms underlying the interaction between DLC1 and various tensins remain controversial.

**Methodology/Principal Findings:**

We used a co-immunoprecipitation assay to identify a previously undocumented binding site at 375–385 of DLC1 that predominantly interacted with the phosphotyrosine binding (PTB) domain of tensin2. DLC1-tensin2 interaction is completely abolished in a DLC1 mutant lacking this novel PTB binding site (DLC1ΔPTB). However, as demonstrated by immunofluorescence and co-immunoprecipitation, neither the focal adhesion localization nor the interaction with tensin1 and C-terminal tensin-like (cten) were affected. Interestingly, the functional significance of this novel site was exhibited by the partial reduction of the RhoGAP activity, which, in turn, attenuated the growth-suppressive activity of DLC1 upon its removal from DLC1.

**Conclusions/Significance:**

This study has provided new evidence that DLC1 also interacts with tensin2 in a PTB domain-dependent manner. In addition to properly localizing focal adhesions and preserving RhoGAP activity, DLC1 interaction with tensin2 through this novel focal adhesion binding site contributes to the growth-suppressive activity of DLC1.

## Introduction

The small, monomeric G-protein Rho has been classically defined as a key biological regulator of the actin cytoskeleton [1]–[3]. In turn, dynamic cytoskeleton turnover controls a wide range of related biological responses, ranging from the definition of cell shape to the promotion of cell migration, cell adhesion and cell spreading [4], [5]. However, an increasing body of evidence suggests that Rho is also involved in controlling important biological functions such as cell proliferation, cell invasion and gene transcription [6]–[11]. Rho is implicated in carcinogenesis, as has been found to be activated in various human cancers [12], [13].

*Deleted in liver cancer 1 (DLC1)* is a tumor suppressor gene located on chromosome 8p21.3-22 and has been shown to be frequently unexpressed in a wide range of human cancers, including hepatocellular carcinoma (HCC) [14]–[23]. *DLC1* encodes a multiple-domain RhoGAP protein with selective activity toward RhoA, B and C and less towards CDC42 but not Rac1 [23], [24]. Extensive studies have shown that DLC1 utilizes this RhoGAP activity to suppress cell proliferation [15], [18], [23], [25]–[29], trigger apoptosis [25] and to reduce cell migration [26], [28], cell invasion and the resultant cancer metastasis in cell lines as well as mouse models with different tissue origins [29]–[31]. In a recent study, the role of DLC1 as a *bona fide* tumor suppressor in HCC was confirmed by a mouse model with a liver-specific, short-hairpin RNA-mediated DLC1 knockdown [32]. Although the role of DLC1 in protecting cells from cancer-related properties has become clear, questions about its biological regulation remain unanswered.

Transcriptionally, *DLC1* expression has been found to be epigenetically silenced in various human cancers. Hypermethylation of the gene promoter region suppressed *DLC1* gene transcription and expression in different tissues [16], [17], [19], [22], [24], [33]–[35]. Post-translationally, rat DLC1 has been shown to be phosphorylated by Akt kinase [36]; however, its occurrence in human DLC1 and its biological significance are still in question. On the other hand, a recent study identified DLC1 mutations in prostate and breast cancers at particular tyrosine and serine residues. These mutations inactivate DLC1 RhoGAP activity through an unknown mechanism [37]. To date, the best characterized regulation of DLC1 at the protein level is its interaction with tensin proteins [27], [28], [38]. Tensins are focal adhesion proteins carrying Src-Homology 2 (SH2) and phosphotyrosine binding (PTB) domains at their C-termini [39]. Accumulating evidence suggests that DLC1 interacts with multiple tensins. In general, DLC1 utilizes an SH2 binding motif involving the residue Y442 to interact with the SH2 domain of tensin1 and C-terminal tensin-like (cten). Mutation at Y442 caused DLC1 to lose its focal adhesion localization and tumor suppressive activity. This observation implies that tensin binding is a key regulatory event in the subcellular localization and the tumor suppressive function of DLC1 [27], [28]. However, we have previously documented interactions between DLC1 and the tensin2 PTB domain [38]. Thus, the mechanism of interaction between DLC1 and various tensins and its biological implications are still controversial.

In the present study, we discovered a novel binding mechanism between DLC1 and tensin2 by identifying an undocumented binding site in DLC1, other than the SH2 binding motif described by others, for interaction with the tensin2 PTB domain. This new site was well conserved in DLC families. We also provide the first evidence for tensin2 interaction as a common characteristic of DLC1 and DLC2. Apart from the binding mechanism, we also demonstrated the functional significance of this novel binding site in regulating the tumor suppressive activity of DLC1.

## Results

### The tensin2 PTB domain was required for DLC1 interaction

**Figure 1:**
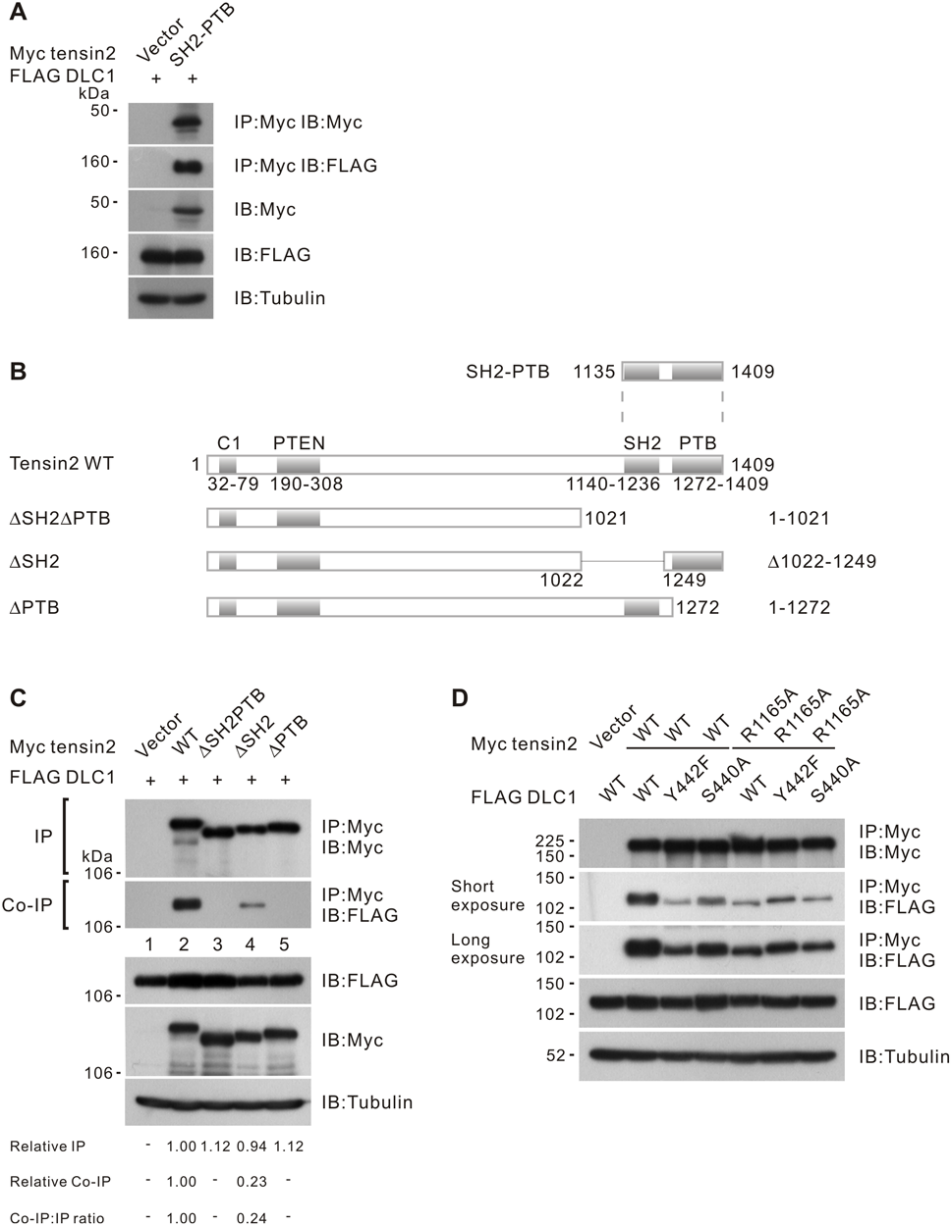
Tensin2 SH2 and PTB domains were responsible for DLC1 binding. (A) Expression of tensin2 SH2-PTB fragment (as described in Materials and Methods and outlined in Fig. 1B) was sufficient for interaction with DLC1. HEK293T cells were transfected with Myc-tagged tensin2 fragment and FLAG-tagged DLC1 constructs as indicated. Cleared cell lysates were incubated with anti-Myc antibody to immunoprecipitate tensin2. DLC1 in the precipitates was detected by immunoblotting with anti-FLAG antibody. (B) Schematic diagram showing the domain structure of the Myc-tagged tensin2 and its C-terminal truncated mutants. The mutants either had both SH2 and PTB domains (ΔSH2ΔPTB) or had individual domains being removed (ΔSH2 and ΔPTB). The SH2-PTB was the tensin2 C-terminus used in Fig. 1A. (C) Mapping the DLC1 binding site in tensin2. HEK293T cells were transfected with Myc-tagged tensin2 and FLAG-tagged DLC1 constructs as indicated. Cleared cell lysates were incubated with anti-Myc antibody to immunoprecipitate tensin2. DLC1 in the precipitates was detected by immunoblotting with anti-FLAG antibody. Removal of the tensin2 SH2 domain (ΔSH2) resulted in the partial reduction of DLC1 binding. Complete loss of DLC1 binding was observed upon the removal of the tensin2 PTB domain (ΔPTB). The band intensity in each lane was measured in the IP and Co-IP panels and the readings were normalized to lane 2. The relative Co-IP-to-IP ratios were also included. (D) Characterization of the binding between tensin2 and focal adhesion localization-defective DLC1 mutants. Wild-type tensin2 or SH2 domain mutant, R1165A, was co-transfected with DLC1 wild-type, Y442F and S440A. The cell lysate was subjected to immunoprecipitation with anti-Myc antibody, followed by immunoblotting with anti-FLAG antibody. Y442F and S440A showed a partial reduction in tensin2 binding, but the SH2 domain mutant, R1165A, did not.

We demonstrated that the C-terminus tensin2 fragment, including the SH2 and PTB domains (SH2-PTB in Fig. 1B), was sufficient to bind DLC1 **(** **Fig. 1A** **)**. To evaluate the importance of individual domains in DLC1 interaction, we prepared several tensin2 deletion mutants including tensin2 ΔSH2ΔPTB, ΔSH2, and ΔPTB and tested their binding affinity towards DLC1 **(** **Fig. 1B** **)**. Tensin2 ΔSH2ΔPTB consistently showed a complete loss of DLC1 binding. In contrast to other reports, we found that removing the SH2 domain in tensin2 only partially reduced DLC1 binding. Interestingly, removing the PTB domain in tensin2 was sufficient to completely abolish the DLC1 interaction, indicating that the PTB domain is required for binding **(** **Fig. 1C** **)**. It has been reported that DLC1 Y442 and S440 form a phospho-independent binding motif for the SH2 domain of tensin1 and cten [27], [28]. We next checked the conservation of these residues in binding to the tensin2 SH2 domain. Interestingly, we found that DLC1 Y442F and S440A mutants showed only a partial reduction in tensin2 binding. To test whether Y442 and S440 mediate the interaction with the tensin2 SH2 domain, we checked the binding affinity between DLC1 and tensin2 R1165A, an SH2 domain mutant with impaired recognition and binding of tyrosine-phosphorylated targets. We found that mutation at R1165A in tensin2 resulted in a drop in tensin2-DLC1 binding. Similar binding affinity toward R1165A was observed among DLC1 wild-type, Y442F and S440A. This indicated that Y442 and S440 could only be effective when the SH2 domain of tensin2 was intact **(** **Fig. 1D** **)**. Collectively, these results support the conclusion that an SH2 domain-mediated mechanism also contributes to DLC1-tensin2 interactions.

### Identification of the tensin2 PTB binding domain in DLC1

**Figure 2:**
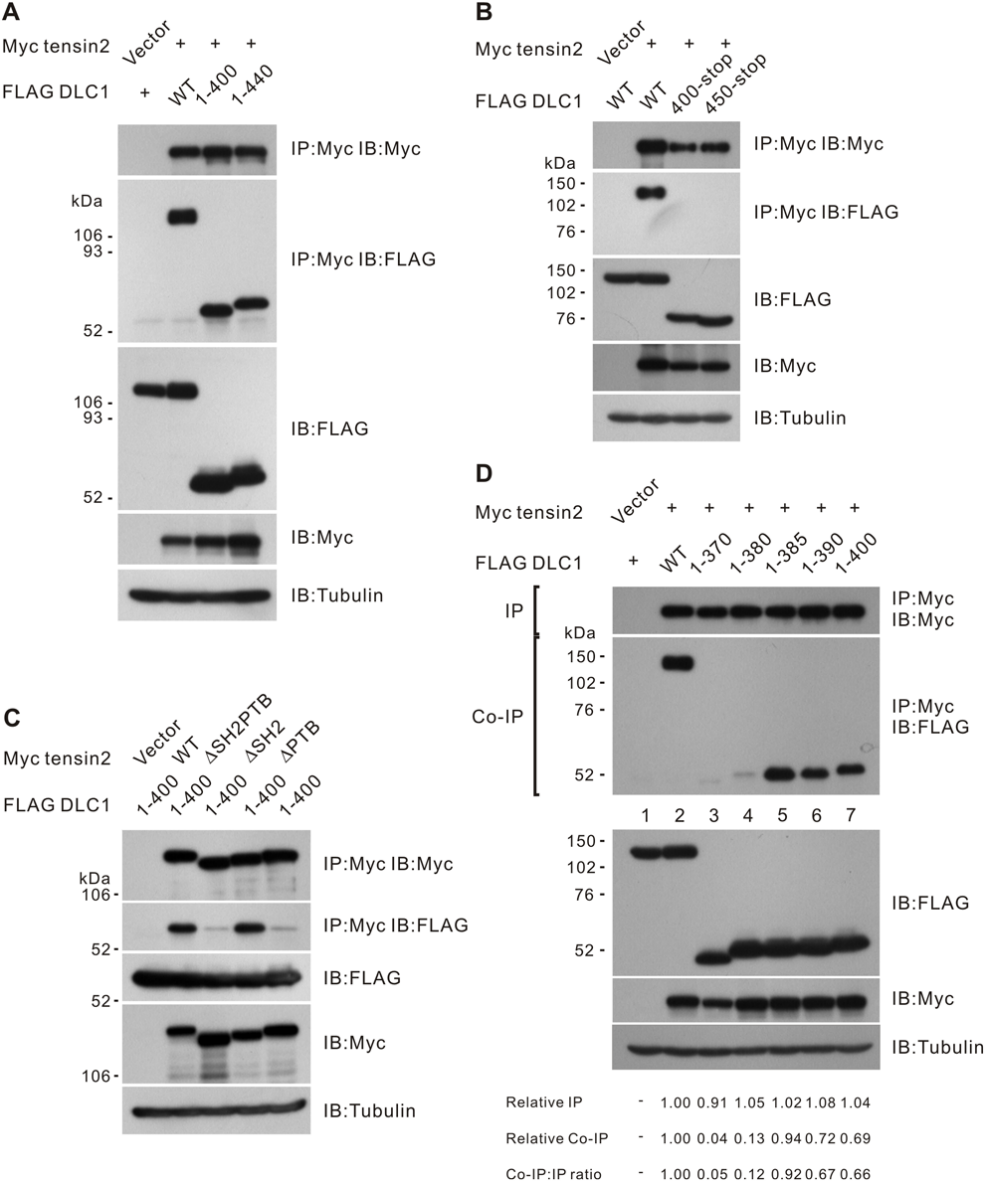
Mapping the tensin2 PTB binding site in DLC1. (A) The N-terminus of DLC1 was sufficient for interaction with tensin2. HEK293T cells were transfected with Myc-tagged tensin2 fragment and FLAG-tagged DLC1 constructs as indicated. Cleared cell lysates were incubated with anti-Myc antibody to immunoprecipitate tensin2. DLC1 in the precipitates was detected by immunoblotting with anti-FLAG antibody. (B) The C-terminus of DLC1 was unable to bind tensin2. Myc-tagged tensin2 was co-transfected with two FLAG-tagged DLC1 C-terminus fragments as indicated. These DLC1 C-terminus fragments could not be co-immunoprecipitated with tensin2. (C) N-terminal fragment 1–400 was involved in tensin2 PTB binding. FLAG-tagged DLC1 1–400 could be co-immunoprecipitated with tensin2. Removal of the tensin2 SH2 domain did not affect the affinity of binding to 1–400, but an intact PTB domain was necessary for the binding. (D) Fine mapping the PTB binding site in DLC1. Myc-tagged tensin2 was co-transfected with a panel of FLAG-tagged DLC1 N-terminus fragments. FLAG-tagged fragments in the precipitates were detected by immunoblotting analysis with anti-FLAG antibody. Fragment 1–385 was the shortest fragment capable of being co-immunoprecipitated with tensin2. The band intensity in each lane was measured in the IP and Co-IP panels and the readings were normalized to lane 2. The relative Co-IP-to-IP ratios were also included.

We next questioned which region of DLC1 was required for this PTB domain-dependent tensin2 interaction. We previously had proposed the center region 375–509 of DLC1 to be the region necessary for tensin2 interaction [38]. To precisely examine this proposed binding region, we cloned and expressed various DLC1 mutants with different truncations created within this region **(** **Fig. 2A** **)**. We found that N-termini of DLC1 1–400 and 1–440, both lacking the reported tensin SH2 binding site (Y442 of DLC1), were sufficient to interact with tensin2. On the other hand, the mutually exclusive C-terminus fragment 400-stop with an intact SH2 binding site was not sufficient to interact with tensin2 **(** **Fig. 2B** **)**. A similar result was observed when 450-stop, which did not contain any predicted tensin binding sites, was used **(** **Fig. 2B** **)**. To provide further evidence that the N-terminus of DLC1 interacted with tensin2 through a PTB domain-dependent mechanism, we checked the interaction between DLC1 1–400 and the aforementioned tensin2 deletion mutants. We observed that the interaction between DLC1 1–400 and tensin2 was not affected, even when the SH2 domain of tensin2 was removed. In contrast, interaction between the two was again abolished when the PTB domain of tensin2 was removed, indicating that this binding was solely PTB domain-dependent **(** **Fig. 2C** **)**. To further pinpoint the PTB binding site in DLC1, we examined the binding of a panel of DLC1 N-terminus fragments to tensin2. Among these fragments, we found that DLC1 1–385 was the shortest fragment that was able to bind tensin2 **(** **Fig. 2D** **)**. Collectively, these results suggest that region DLC1 375–385 functions as a tensin2 PTB binding site and is required for tensin2 binding.

### Conservation of tensin2 PTB binding domain in DLC2

**Figure 3:**
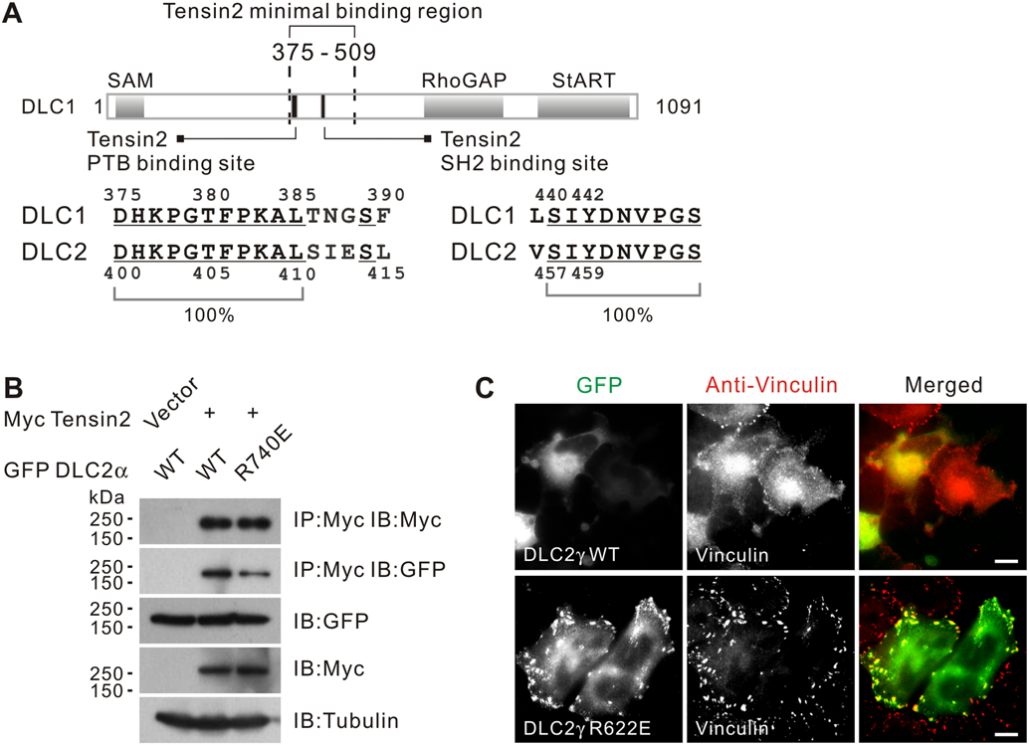
Conservation of the tensin2 PTB binding site in DLC1/2. (A) Schematic showing the tensin2 SH2 and PTB binding sites in DLC1. Within the minimal tensin2 binding region 375–509, separate elements are involved in mediating tensin2 binding via PTB or SH2 mechanisms. These elements are well conserved in the DLC1 paralog, DLC2. Conserved residues were underlined. (B) Interaction between DLC2 and tensin2 in a RhoGAP-independent manner. HEK293T cells were transfected with Myc-tagged tensin2 and GFP-tagged DLC2 constructs as indicated. Cleared cell lysates were incubated with anti-Myc antibody to immunoprecipitate tensin2. DLC2 in the precipitates was detected by immunoblotting with anti-GFP antibody. (C) GFP-DLC2γ showed focal adhesion localization in a RhoGA-independent manner. Expression of GFP-DLC2γ induced severe cell shrinkage when expressed in SMMC-7721 cells. Focal adhesion localization of the DLC2γ RhoGAP mutant R622E was detected. The endogenous vinculin was visualized by anti-vinculin antibody. Scale bar = 10 µm.

To check the biological conservation of the mapped tensin2 binding regions in other DLC family members, we performed amino acid alignment between DLC1 and DLC2 [40], [41]. We found that both the SH2 and PTB binding sites are well conserved in DLC2. Corresponding serine (S457) and tyrosine (Y459) residues in the SH2 binding domain were found in DLC2. The PTB binding site in DLC1 375–385 was found to match with DLC2 400–410, suggesting a potential role of this region in mediating PTB domain-dependent binding between DLC family members and tensin2 **(** **Fig. 3A** **)**. Based on the conservation of tensin binding sites in DLC2, we hypothesized that DLC2 might interact with tensin2. Using co-immunoprecipitation, we demonstrated that DLC2α and tensin2 do interact, which confirmed our speculation. As with DLC1, the RhoGAP activity of DLC2 was not required for tensin2 interaction, as shown by the positive interaction of the DLC2 RhoGAP mutant, R740E, with tensin2 **(** **Fig. 3B** **)**. We questioned whether DLC2 also localized to focal adhesions in SMMC-7721 cells. We found that expression of DLC2γ induced severe cell shrinkage. To facilitate the observation, we instead used a functionally inactive mutant, DLC2γ R622E, that harbored a mutation in its RhoGAP domain [41]. We found that DLC2γ R622E could co-localize with vinculin **(** **Fig. 3C** **)**, indicating that other DLC proteins may also localize to focal adhesions.

### DLC1ΔPTB mutant showed specific loss of tensin2 binding but preserved its focal adhesion localization

**Figure 4:**
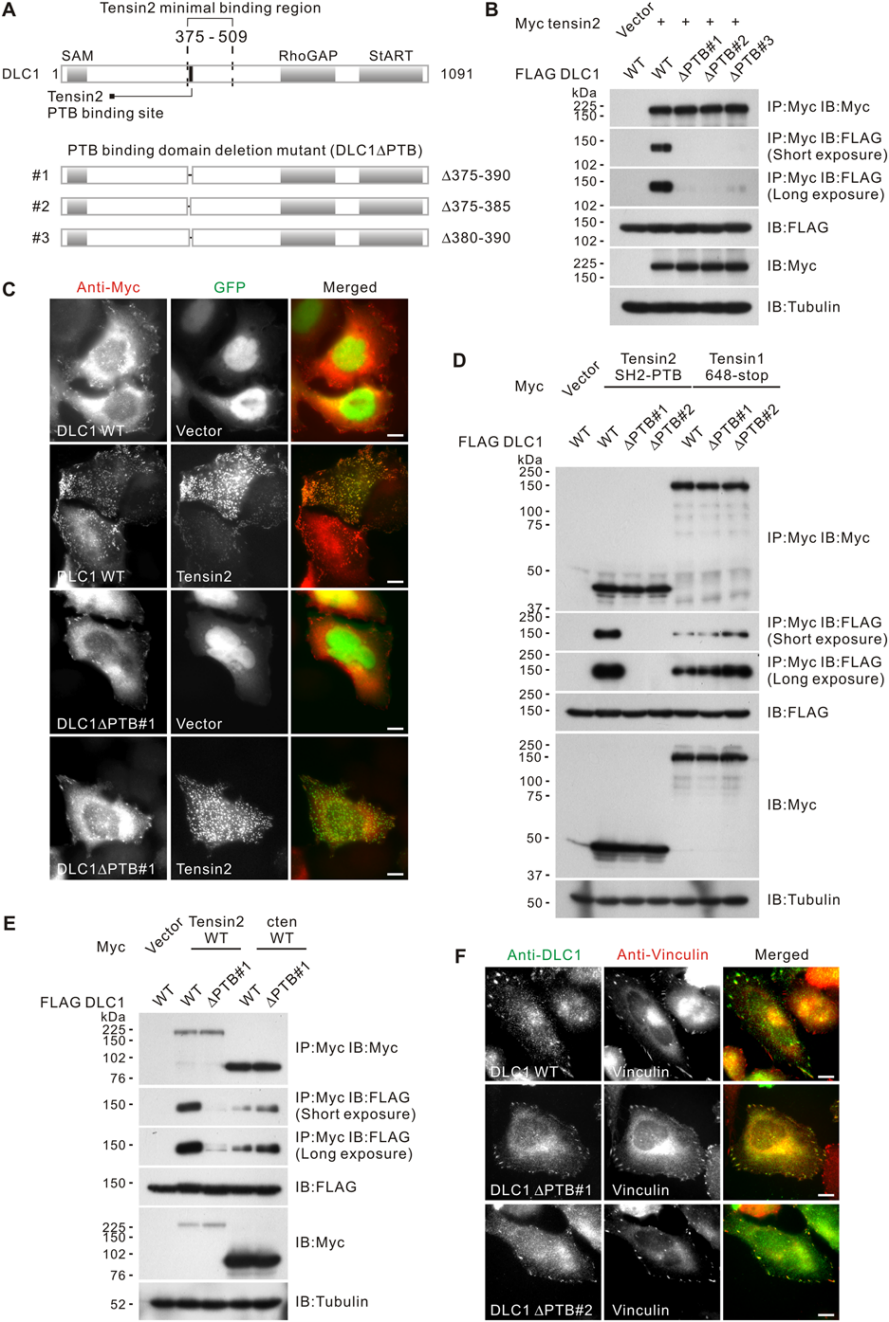
Characterization of the DLC1ΔPTB mutants. (A) Schematic showing the structure of three FLAG-tagged DLC1ΔPTB mutants: Δ375–390, Δ375–385 and Δ380–390 (ΔPTB#1–3). (B) DLC1ΔPTB showed reduced tensin2 binding. HEK293T cells were transfected with Myc-tagged tensin2 and FLAG-tagged DLC1 constructs as indicated. Cleared cell lysates were incubated with anti-Myc antibody to immunoprecipitate tensin2. DLC1 in the precipitates was detected by immunoblotting analysis with anti-FLAG antibody. Removal of the PTB binding domain in DLC1 resulted in loss of tensin2 binding. (C) DLC1ΔPTB showed reduced co-localization with tensin2. SMMC-7721 cells were transiently co-transfected with GFP vector or GFP-tensin2 and the indicated Myc-tagged DLC1. DLC1 was visualized by using anti-Myc antibody, followed by Texas Red-conjugated secondary antibody. Scale bar = 10 µm. (D) DLC1ΔPTB could interact with tensin1. HEK293T cells were transfected with Myc-tagged tensin2 SH2-PTB (as outlined in Fig. 1B) or Myc-tagged tensin1 C-terminus 648-stop (as outlined in Fig. S1) and FLAG-tagged DLC1 constructs as indicated. Cleared cell lysates were incubated with anti-Myc antibody to immunoprecipitate tensin2 or tensin1. DLC1 in the precipitates was detected by immunoblotting analysis with anti-FLAG antibody. Removal of the PTB binding domain in DLC1 did not affect the tensin1 binding. (E) DLC1ΔPTB could interact with cten. HEK293T cells were transfected with Myc-tagged tensin2 or Myc-tagged cten and FLAG-tagged DLC1 constructs as indicated. Cleared cell lysates were incubated with anti-Myc antibody to immunoprecipitate tensin2 or cten. DLC1 in the precipitates was detected by immunoblotting analysis with anti-FLAG antibody. Removal of the PTB binding domain in DLC1 did not affect the cten binding. (F) DLC1ΔPTB showed focal adhesion localization in SMMC-7721 cells. SMMC-7721 cells were transiently co-transfected with GFP-tensin2 and the FLAG-tagged DLC1 as indicated. DLC1 was visualized by using anti-DLC1 antibody, followed by FITC-conjugated secondary antibody. The endogenous vinculin was visualized by anti-vinculin antibody, followed by Texas Red-conjugated antibody. Scale bar = 10 µm.

To study the role of the tensin2 PTB binding domain of DLC1, we cloned three separate DLC1 mutants that had minimal alterations to the following structural elements: Δ375–390 (ΔPTB#1), Δ375–385 (ΔPTB#2) and Δ380–390 (ΔPTB#3), each carrying a specific internal deletion within the PTB domain (DLC1ΔPTB) **(** **Fig. 4A** **)**. To confirm the role of the PTB domain in tensin2 binding, we first tested the binding of these mutants with tensin2. With a co-immunoprecipitation assay, we found that all DLC1ΔPTB mutants showed a loss of tensin2 binding **(** **Fig. 4B** **)**. The loss of binding was further confirmed by decreased co-localization between DLC1ΔPTB#1 and tensin2 **(** **Fig. 4C** **)**. To address the specificity of the mapped PTB binding motif toward other tensin proteins, we performed a co-immunoprecipitation assay using a C-terminus fragment of tensin1, 648-stop. Tensin1 648-stop covers the C-terminus SH2 and PTB domains, which have been shown to be important in DLC1 binding [28]. We have confirmed their role in DLC1 interaction with our co-immunoprecipitation system **(Fig. S1)**. Interestingly, we found that tensin1 648-stop showed comparable binding affinity with DLC1 wild-type and DLC1ΔPTB **(** **Fig. 4D** **)**. A positive interaction with DLC1 was also observed when cten was immunoprecipitated in the co-immunoprecipitation assay **(** **Fig. 4E** **)**. These observations support the specific involvement of this PTB binding motif with tensin2. To address whether the removal of the PTB domain would affect the focal adhesion localization of DLC1, we studied the localization of the DLC1ΔPTB in SMMC-7721 HCC cells with immunofluorescence. We found that DLC1ΔPTB mutants maintained their focal adhesion localization, as indicated by their co-localization with the focal-adhesion-associated protein vinculin. This observation suggested that removal of the PTB binding site in DLC1 affects its binding with tensin2 but preserves its focal adhesion localization, possibly via interaction with other tensins through a PTB-independent binding mechanism **(** **Fig. 4F** **)**.

### DLC1ΔPTB showed partial reduction in RhoGAP activity but was sufficient to suppress actin stress fiber formation

**Figure 5:**
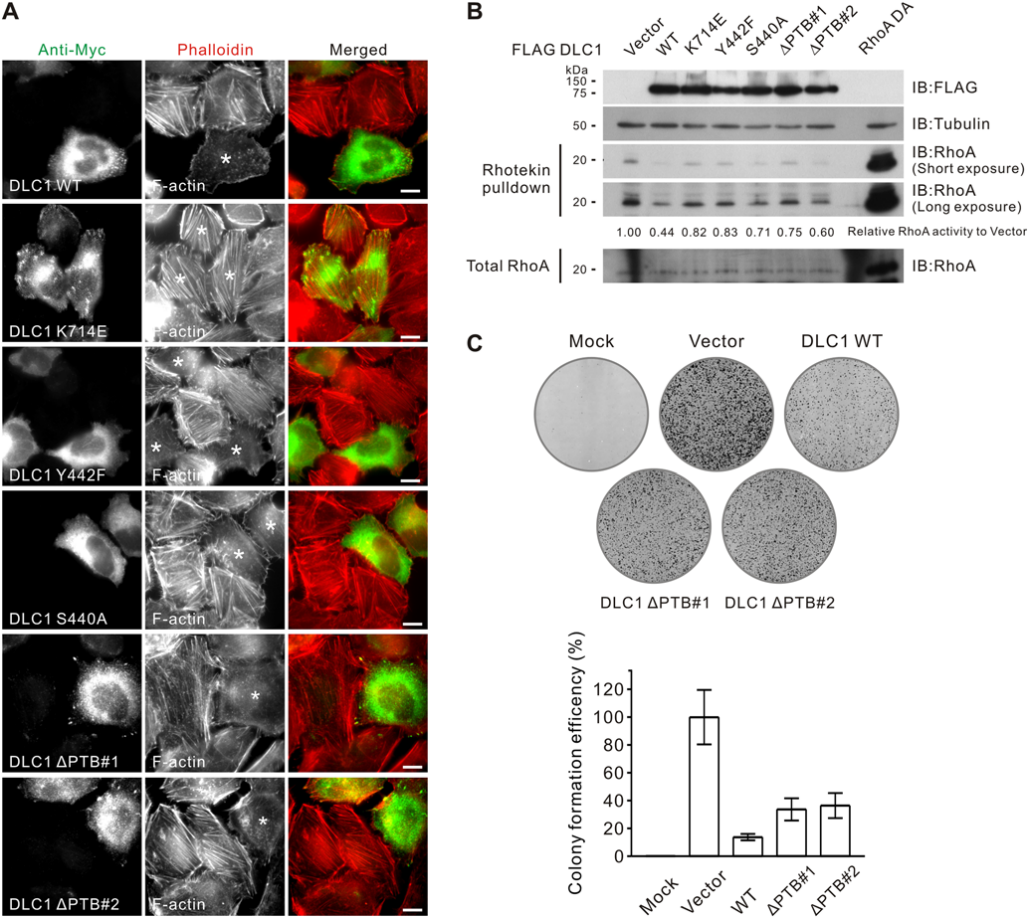
Tensin2 PTB binding was not necessary for DLC1 to suppress the formation of actin stress fibers but was required to suppress colony formation. (A) SMMC-7721 HCC cells were transfected with the indicated Myc-tagged DLC1 constructs and the actin stress fibers were stained with TRITC-conjugated phalloidin 1 hour after the serum induction. The asterisk (*) marks the DLC1-transfected cells. DLC1 Y442F, S440A, ΔPTB#1 and #2 could suppress actin stress fiber formation as efficiently as DLC1 WT. Scale bar = 10 µm. (B) HEK293T cells were transfected with the indicated FLAG-tagged DLC1 plasmid for 24 hours. After transfection, the cells were serum-starved for 24 hours and stimulated with 5 µM Lysophosphatidic acid for 30 minutes. Cell lysate was then collected and subjected to GST-RBD pull-down for RhoA-GTP. The pull-down samples were subjected to SDS-PAGE analysis and immunodetected with anti-RhoA antibodies. The band intensity of the active RhoA in each lane was measured and the readings were normalized to the vector-transfected cells. The DLC1, tubulin and RhoA in total cell lysate were immunodetected with anti-FLAG, anti-tubulin and anti-RhoA antibodies, respectively. (C) HeLa cells were transfected with the indicated DLC1 constructs and selected with G418 for 2 weeks. The colonies formed were visualized by crystal violet staining. The mean difference in colony formation efficiency between groups was found to be statistically significant (*p*<0.001; one-way ANOVA test).

Active RhoA is best known for its role in simulating actin stress fiber formation. Being a negative regulator of RhoA, DLC1 suppresses actin stress fiber formation in a RhoGAP-dependent manner [26]. Using actin stress fibers as an indirect biological readout, we asked whether the RhoGAP activity of DLC1ΔPTB mutants would be affected. We found that DLC1ΔPTB mutant-transfected cells showed reduced actin stress fiber formation when compared with the neighboring non-transfected cells. This observation was similar to those of the focal adhesion localization-defective mutants of DLC1, Y442F and S440A, each of which carries a point mutation in the tensin SH2 binding domain **(** **Fig. 5A** **)**. To directly quantify the RhoA inactivation, we performed a Rhotekin pull-down assay to determine the Rho-GTP activity levels in cells expressing different DLC1 mutants. Although we found that the DLC1ΔPTB mutant could suppress Rho-GTP activity, it was interesting that this suppression was less effective when compared with that of wild-type DLC1 **(** **Fig. 5B** **)**. On the other hand, it is noted that the Y442F and S440A mutants also showed a reduction in suppressing Rho-GTP activity levels. Altogether, we found that the DLC1ΔPTB mutant showed a partial reduction in intrinsic RhoGAP activity, but that this activity was sufficient to suppress actin stress fiber formation.

### DLC1ΔPTB showed reduced growth-suppressing activity

The ability of DLC1 to suppress tumor growth has been well documented. We questioned whether the aforementioned DLC1ΔPTB mutants displayed any difference in DLC1-induced growth suppression. In a HeLa cell colony formation assay, wild-type DLC1 significantly suppressed colony formation when compared with the vector control. In contrast, ectopic expression of DLC1ΔPTB mutants resulted in a significantly increased number of colonies when compared with wild-type DLC1 **(** **Fig. 5C** **)**. This observation indicates that even when DLC1ΔPTB shows proper focal adhesion localization, loss of this tensin2 PTB binding motif will result in reduced growth suppression, probably due to the partial reduction in RhoGAP activity. It is likely that proper tensin2 PTB binding contributes to DLC1-induced growth suppression.

## Discussion

DLC1 has been shown to exert biological functions resembling those of classical tumor suppressor genes. The diverse tumor-suppressive effects of DLC1 are strongly dependent on the presence of a functional RhoGAP domain [26], [28]. However, several studies have shown that RhoGAP activity alone is not sufficient for its tumor-suppressive function [26]–[28], [42]. Previous structural analysis from our group provided the first evidence that these functions of DLC1 could be restored only when the region between the SAM and RhoGAP domains was included [26]. The identification of tensin2 as the first binding protein that interacts with this functionally important region further hinted at the relationship between tensin2 interaction and DLC1 function [38]. This notion was further supported by the discovery of cten and tensin1 as binding partners of DLC1 in subsequent studies, implying that it is biologically important for tensin family proteins to work co-operatively with DLC1 [27], [28].

We previously proposed that DLC1 375–509 is the minimal binding region of tensin2. This region covers the key residues Y442 and S440, which were suggested by other studies to constitute an SH2 binding site for cten and tensin1 [27], [28]. However, we could not provide direct evidence that the tensin2 SH2 domain was sufficient for DLC1 binding [38]. In this study and our previous report, our findings consistently showed that that tensin2 PTB, rather than SH2, domain directly interacted with DLC1.

There are a few possible explanations for the discrepant findings. First, although the SH2 and PTB domains in tensin proteins have a high sequence homology, differences in their sequences may result in different binding mechanisms for DLC1 and other tensin family members. Second, different binding assays were used by different groups to study the mechanism of binding between tensin and DLC1. For instance, yeast-based binding and GST pull-down assays were used by Liao et al. and Qian et al., respectively, while *in vivo* co-immunoprecipitation was employed in our present study. Third, C-terminus fragments of tensin that contained either one or both of the SH2 and PTB domains were used in mapping the DLC1 binding site in the studies by Liao et al. and Qian et al. This experimental design presumed that the N-terminus region of the tensin protein neither was involved in binding nor contributed to the normal protein folding of the C-terminus region.

**Figure 6:**
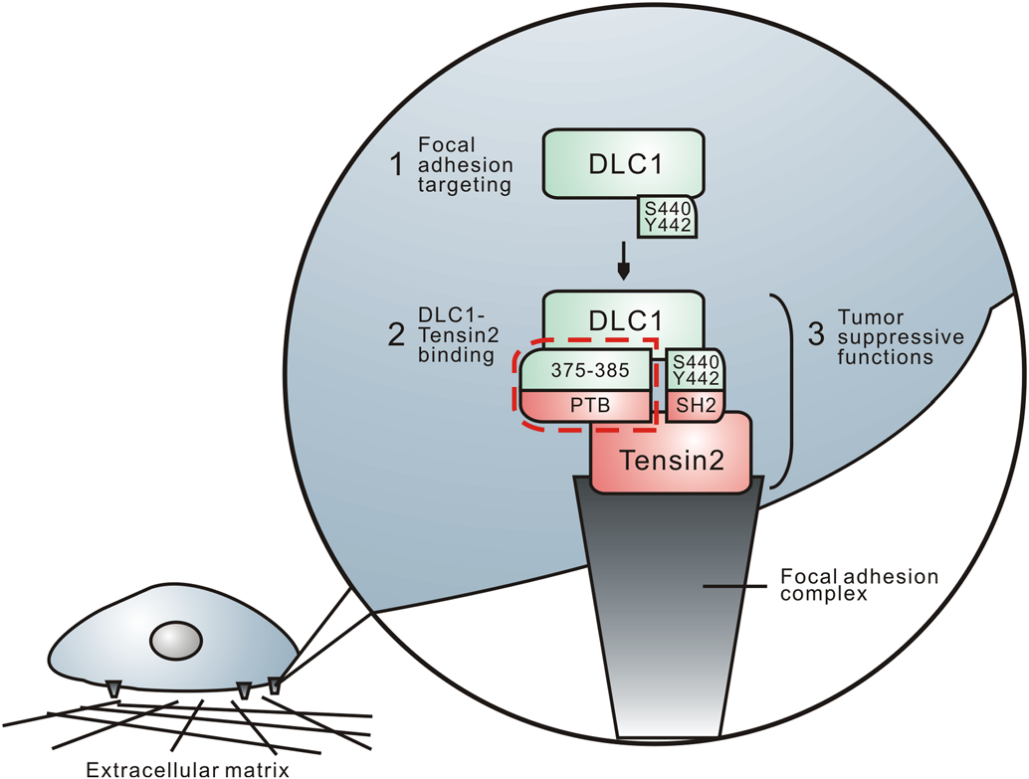
Proposed model of the focal adhesion targeting and tensin2 interaction of DLC1. DLC1 was targeted to the focal adhesions and required residues Y442 and S440. At the focal adhesion, besides the SH2 binding mechanism, DLC1 utilized region 375–385 to interact with the PTB domain of tensin2, as indicated by the dotted line. The formation of this binding complex was important for DLC1 to exert growth-suppressive function.

In the present study, we provide the first evidence that specific removal of the SH2 and PTB domains in tensin2 affects DLC1 binding to different extents **(** **Fig. 1C** **)**. To provide evidence that the PTB domain plays a critical role in DLC1-tensin2 binding, we mapped the PTB-binding domain at the N-terminus of DLC1 **(** **Fig. 2** **)** and confirmed its role by characterizing the DLC1ΔPTB internal deletion mutants **(** **Fig. 4A** **)**. We identified an undocumented region 375–385 of DLC1 as the PTB-domain-binding site and the DLC1-tensin2 interaction was lost when this region was removed **(** **Fig. 4B** **)**. It is well established that the PTB domain recognizes a NPXY motif, as illustrated in its binding with integrin β [43]. However, this motif was not found in our mapped region in DLC1, implying that the mode of PTB-mediated interaction is a probably atypical. Although DLC1ΔPTB showed loss of tensin2 binding, it was still able to localize to focal adhesions **(** **Figs. 4C and 4F** **)**. In addition, we found that the PTB domain was necessary for the focal adhesion localization of tensin2 (data not shown). Thus, in addition to acting as the physical binding site for DLC1, the PTB domain may also be important for bringing tensin2 in close proximity to DLC1 at focal adhesions and facilitating their interaction. Collectively, these results suggest that DLC1 utilizes region 375–385 in mediating tensin2 binding, while it utilizes Y442 and S440 residues for focal adhesion targeting **(** **Fig. 6** **)**.

It is currently unknown whether the proposed tensin PTB binding domain in DLC1 is involved in binding with other tensins. This remains to be investigated. There have been reports that expression of the tensin1 PTB domain is sufficient for interaction with DLC1. Also, deleting the whole tensin SH2 binding motif (440–448), instead of introducing a Y442F point mutation in DLC1, is sufficient for interaction with tensin1 [28]. This indicates that PTB-dependent binding may be present between DLC1 and tensin1, but plays a subtle role, unlike the DLC1 and tensin2 interaction. Based on our present findings, we conclude that the PTB binding mechanism is tensin2-specific **(** **Figs. 4B, 4D and 4E** **)**, whereas other tensins utilize an SH2 binding mechanism [27], [28]. Possible compensation by other tensins may explain why DLC1ΔPTB can be localized to focal adhesions in the absence of tensin2 interactions. In addition, we found that DLC1ΔPTB showed a partial reduction in RhoGAP activity, which resulted in a partial reduction in growth suppression **(** **Figs. 5B and 5C** **)**.

A limitation in our present study is the use of ectopically expressed tensin2. Detection of endogenous tensin2 was not possible, as the tensin2 antibody was not available in our laboratory. Interaction between endogenous DLC1 and tensin2 must be examined to reflect their binding in the actual biological context. On the other hand, we performed preliminary quantitative real time PCR analysis to determine the mRNA expression levels of DLC1 and tensin2 in human HCC tissues. Our unpublished data showed that underexpression of both DLC1 and tensin2 was correlated with shorter overall survival when compared with those who had normal expression of either or both genes, supporting the possible functional association of DLC1-tensin2 with hepatocarcinogenesis.

Altogether, we have identified a novel tensin2 PTB binding site in DLC1 and demonstrated its involvement in tensin2 interactions. Although the removal of the PTB binding site did not affect the focal adhesion localization, it partially reduced the RhoGAP activity of DLC1, which attenuated its growth suppressive function. It would be interesting to uncover how DLC1 may control the activity of other focal adhesion molecules. We have also provided early evidence that the DLC1 paralog, DLC2, may also interact with tensin2 and localize to focal adhesions **(** **Figs. 3B and 3C** **)**. The conservation of the tensin binding site in DLC2 warrants further investigation into the localization control of the other DLC family members. This will clearly help to determine if DLC members are biologically redundant or if they have different compartmentalization for performing separate biological functions.

## Materials and Methods

### Plasmids

DNA expression constructs using pCS2+MT, pEGFP-C1 (BD Biosciences Clontech, Palo Alto, CA) and FLAG-pcDNA3.1(+) vectors were prepared by standard molecular cloning techniques and PCR amplification of the described fragments. Eukaryotic expression vectors for Myc-tagged proteins were derived from pCS2+MT and prepared as follows: DLC1 1–1091 (WT, wild-type), K714E (RhoGAP mutant), Y442F, S440A, Δ375–390 (ΔPTB#1), Tensin2 1–1409 (WT, wild-type), 1–1021 (ΔSH2ΔPTB), Δ1022–1272 (ΔSH2), 1–1272 (ΔPTB), 1135–1409 (SH2-PTB), R1165A, tensin1 1–1186, 648-stop and cten. cten cDNA was amplified by PCR using pEGFP-C1-cten as the template (a gift kindly provided by Professor Yosef Yarden from the Weizmann Institute of Science, in Israel). The GFP-tagged expression vector, pEGFP-C1, carrying DLC1 WT, ΔPTB1 and Δ375–385 (ΔPTB#2), was constructed. GFP-tagged DLC2α, DLC2αR740E, DLC2γ, DLC2γ R622E were prepared as previously described [41]. FLAG-tagged eukaryotic expression vectors were derived from FLAG-pcDNA3.1(+) and prepared as follows: DLC1 FL, ΔPTB#1, ΔPTB#2, Δ380–390 (ΔPTB#3), 1–370, 1–380, 1–385, 1–390, 1–400,1–440, 400-stop, 450-stop. All of the DNA expression constructs were confirmed by DNA sequencing.

### Cell culture and transfection

Human embryonic kidney cells, HEK293T, and a human cervical carcinoma cell line, HeLa, were obtained from American Type Culture Collection (Manassas, VA), whereas the human HCC cell line SMMC-7721 was obtained from the Shanghai Institute of Cell Biology. HEK293T and SMMC-7721 were cultured in DMEM high-glucose medium supplemented with 10% (v/v) fetal bovine serum, penicillin, and streptomycin at 37°C in a humidified incubator with 5% CO_2_ in air. HeLa cells were cultured in DMEM low-glucose medium supplemented with 10% (v/v) fetal bovine serum, penicillin and streptomycin under the same conditions. Transfection with the indicated plasmid was done with Lipofectamine 2000 reagent, according to the manufacturer's instructions (Invitrogen, Carlsbad, CA).

### Cell lysis, co-immunoprecipitation and western blotting

Transfected HEK293T cells were lysed with NET-N buffer (25 mM Tris-HCl, pH 8.0, 50 mM NaCl, 0.2 mM EDTA, 0.1% NP40) and a cocktail of protease inhibitors (Roche, Mannheim, Germany) on ice for 20 minutes. The cell lysate was cleared by centrifugation at 16,000 g for 15 minutes at 4°C. Seven- to eight-hundred micrograms of cell lysate were incubated with 2 µg of anti-Myc antibody (Santa Cruz Biotechnology, Santa Cruz, CA) with gentle rotation at 4°C overnight. The antibody complex was collected by incubation with protein-A sepharose (Amersham/GE Healthcare Bio-Sciences, Piscataway, NJ) for 5 hours at 4°C. The protein-A sepharose was then washed 5 times with NET-N buffer and the boiled immunoprecipitates were subjected to SDS-PAGE analysis. The Myc-tagged and FLAG-tagged proteins were immunoblotted with anti-Myc (Santa Cruz Biotechnology) and anti-FLAG (Sigma, St. Louis, MO) antibodies, respectively. The endogenous tubulin was detected by anti-tubulin antibody (Sigma) as a loading control. The band intensity in the western blot was measured by AlphaEase FC Software (Alpha Innotech Corporation, San Leandro, CA).

### Immunofluorescence microscopy

For the subcellular localization studies, SMMC-7721 cells seeded on coverslips were transfected with specific DLC1 and/or tensin2 constructs. The transfected cells were fixed with 4% paraformaldehyde in PBS for 15 minutes and then permeabilized with 0.2% Triton-X-100 in PBS for 10 minutes, followed by blocking with 3% bovine serum albumin in PBS for 20 minutes at room temperature. The blocked coverslips were then incubated with the primary antibody, followed by FITC- or Texas Red-conjugated secondary antibody, for 1 hour each. Myc-tagged protein was stained with anti-Myc antibody (Santa Cruz Biotechnology). FLAG-tagged DLC1 was stained with anti-DLC1 antibody. The endogenous vinculin was stained by anti-vinculin antibody (Sigma). The F-actin was visualized by tetramethylrhodamine B isothiocyanate (TRITC)-labeled phalloidin (Sigma) one hour after serum induction. The processed coverslips were mounted in Vectashield anti-fade mounting medium (Vector Laboratories, Burlingame, CA). Images were captured by a Leica Q550CW fluorescence microscope (Leica, Wetzler, Germany).

### Rhotekin pull-down assay

HEK293T cells were transfected with 4 µg of FLAG-tagged DLC1 construct. The RhoA activity level in DLC1 transfected cells was detected using a Rho Activation Assay Biochem Kit (Cytoskeleton) as described by Liao et al [37]. Two µg of dominant active RhoA (RhoA DA) in pcDNA3.1(−) was transfected as a positive control, as previously described [41].

### Colony formation assay

HeLa cells seeded at 1×10^5^ cells per well on a 6-well plate were transfected with 2 µg GFP-tagged DLC1 construct for 24 hours. Transfected cells were then seeded onto a new 6-well plate in a 1∶5 dilution and selected with 0.5 mg/ml G418. The colony was fixed two weeks after selection and visualized by crystal violet staining. The number of colonies formed was analyzed by AlphaEase FC Software (Alpha Innotech Corporation).

## Supporting Information

Figure S1 C-terminus of tensin1 with SH2 and PTB domain was required for DLC1 interaction. (A) Schematic outlining the structures of the tensin1 N-terminus fragment 1–1186 and the tensin1 C-terminus fragment 648-stop, with respect to the full length. (B) HEK293T cells were transfected with Myc-tagged tensin1 and FLAG-tagged DLC1 constructs as indicated. Cleared cell lysates were incubated with anti-Myc antibody to immunoprecipitate tensin1. DLC1 in the precipitates was detected by immunoblotting with anti-FLAG antibody. Removal of the tensin1 SH2 and PTB at the C-terminus completely abolished DLC1 interaction.
(0.44 MB TIF)
