# Supplementary figures and images for: Deleted in Liver Cancer 1 (DLC1) Utilizes a Novel Binding Site for Tensin2 PTB Domain Interaction and Is Required for Tumor-Suppressive Function
Source: PLoS One. 2009 May 15;4(5):e5572. doi: 10.1371/journal.pone.0005572 (PMC2680019; doi:10.1371/journal.pone.0005572)

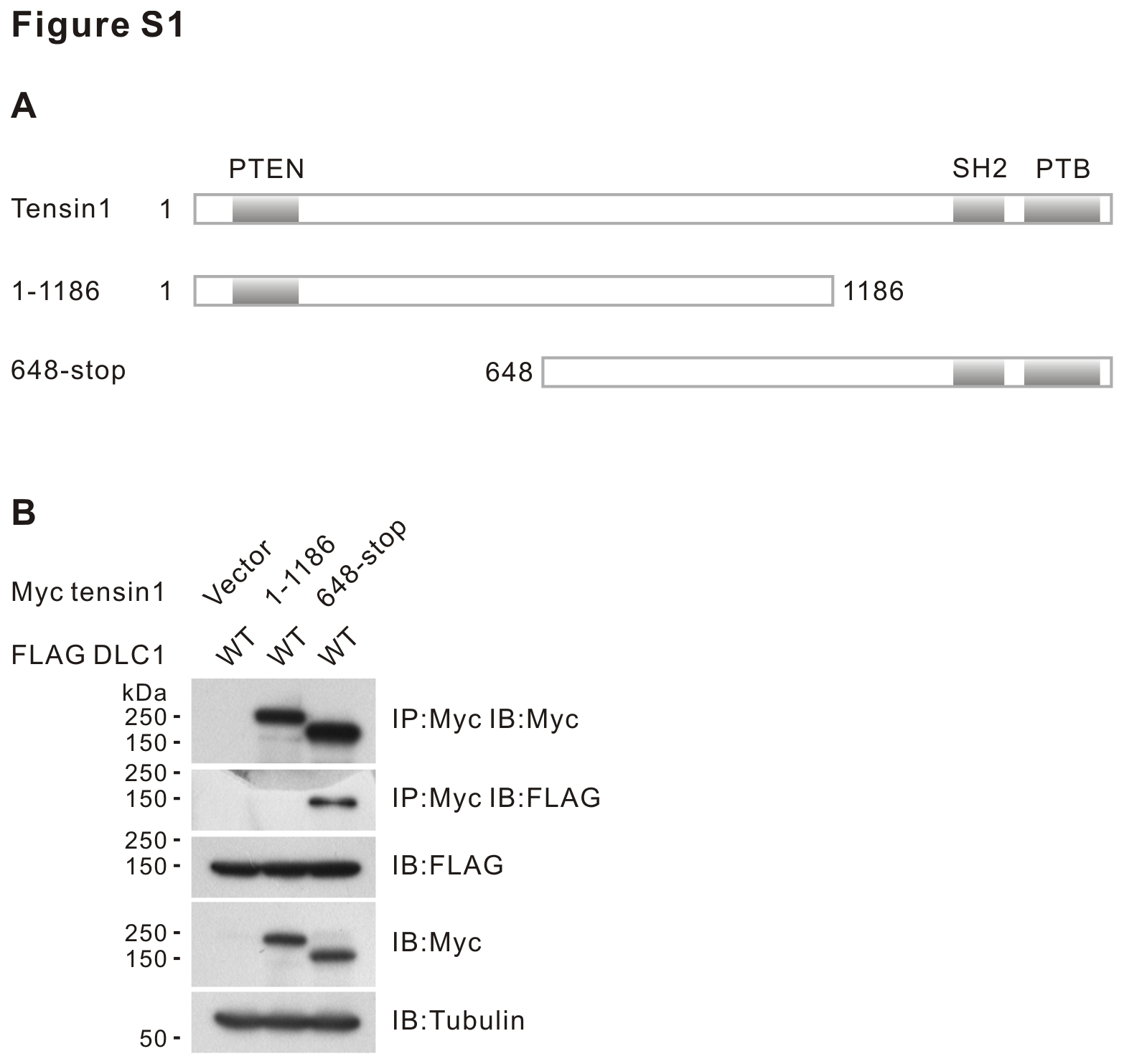

Supplement: Figure S1 — C-terminus of tensin1 with SH2 and PTB domain was required for DLC1 interaction. (A) Schematic outlining the structures of the tensin1 N-terminus fragment 1–1186 and the tensin1 C-terminus fragment 648-stop, with respect to the full length. (B) HEK293T cells were transfected with Myc-tagged tensin1 and FLAG-tagged DLC1 constructs as indicated. Cleared cell lysates were incubated with anti-Myc antibody to immunoprecipitate tensin1. DLC1 in the precipitates was detected by immunoblotting with anti-FLAG antibody. Removal of the tensin1 SH2 and PTB at the C-terminus completely abolished DLC1 interaction. (0.44 MB TIF) [file pone.0005572.s001.tif]
